# *Mycobacterium tuberculosis* WhiB3 Responds to Vacuolar pH-induced Changes in Mycothiol Redox Potential to Modulate Phagosomal Maturation and Virulence*[Fn FN2]

**DOI:** 10.1074/jbc.M115.684597

**Published:** 2015-12-04

**Authors:** Mansi Mehta, Raju S. Rajmani, Amit Singh

**Affiliations:** From the ‡ Department of Microbiology and Cell Biology, Centre for Infectious Disease Research, Indian Institute of Science, Bangalore 12, India and; the § International Centre for Genetic Engineering and Biotechnology, New Delhi 67, India

**Keywords:** lysosomal acidification, Mycobacterium tuberculosis, pH regulation, redox signaling, virulence factor

## Abstract

The ability of *Mycobacterium tuberculosis* to resist intraphagosomal stresses, such as oxygen radicals and low pH, is critical for its persistence. Here, we show that a cytoplasmic redox sensor, WhiB3, and the major *M. tuberculosis* thiol, mycothiol (MSH), are required to resist acidic stress during infection. WhiB3 regulates the expression of genes involved in lipid anabolism, secretion, and redox metabolism, in response to acidic pH. Furthermore, inactivation of the MSH pathway subverted the expression of *whiB3* along with other pH-specific genes in *M. tuberculosis*. Using a genetic biosensor of mycothiol redox potential (*E_MSH_*), we demonstrated that a modest decrease in phagosomal pH is sufficient to generate redox heterogeneity in *E_MSH_* of the *M. tuberculosis* population in a WhiB3-dependent manner. Data indicate that *M. tuberculosis* needs low pH as a signal to alter cytoplasmic *E_MSH_*, which activates WhiB3-mediated gene expression and acid resistance. Importantly, WhiB3 regulates intraphagosomal pH by down-regulating the expression of innate immune genes and blocking phagosomal maturation. We show that this block in phagosomal maturation is in part due to WhiB3-dependent production of polyketide lipids. Consistent with these observations, *Mtb*Δ*whiB3* displayed intramacrophage survival defect, which can be rescued bypharmacological inhibition of phagosomal acidification. Last, *Mtb*Δ*whiB3* displayed marked attenuation in the lungs of guinea pigs. Altogether, our study revealed an intimate link between vacuolar acidification, redox physiology, and virulence in *M. tuberculosis* and discovered WhiB3 as crucial mediator of phagosomal maturation arrest and acid resistance in *M. tuberculosis*.

## Introduction

*Mycobacterium tuberculosis* causes a chronic persistent infection that affects one-third of the world's population (see the World Health Organization website). Macrophage is the major human host cell for growth, survival, and persistence of *M. tuberculosis*. Studies indicate that *M. tuberculosis* continuously senses the phagosomal environment and modulates genetic pathways to regulate intramacrophage growth for long term persistence (1–3). In this regard, acidic pH has recently been appreciated as an important intraphagosomal signal sensed by *M. tuberculosis* to regulate gene expression and establish chronic infection (3). The importance of pH emerged from several studies showing that pathogenic mycobacteria successfully restrict fusion of phagosomes with acidic lysosomes and therefore multiply in a growth-permissive vacuolar compartment with a pH of ∼6.2 (4–6). However, upon activation of macrophages by interferon-γ (IFN-γ) and *Escherichia coli*-derived lipopolysaccharide (LPS), the pH of the *M. tuberculosis* phagosome drops to <5.0, resulting in *M. tuberculosis* growth restriction (6–8). Hence, the inhibition of phagosomal maturation during early stages of infection and induction of acid resistance mechanisms later during immune-activation are considered major virulence strategies adopted by *M. tuberculosis* to establish chronic infection (9). Despite the recognized role of acidic pH in regulating *M. tuberculosis* pathogenesis, the mechanisms by which *M. tuberculosis* responds to fluctuations in phagosomal pH and calibrates its gene expression for intracellular growth and persistence remain poorly characterized.

Recent studies indicate that *M. tuberculosis* maintains a neutral intrabacterial pH (∼7.2) after exposure to a range of acidic pH levels (from 6.2 to 4.5) *in vitro*, and inside the acidic phagosomal milieu of resting or activated macrophage (10). This indicates that *M. tuberculosis* stably maintains intrabacterial pH homeostasis during infection and, therefore, that the intrabacterial pH *per se* is unlikely to be the signal that triggers alterations in *M. tuberculosis* gene expression in response to changes in phagosomal acidity. Therefore, novel insights are needed to discover which aspects of mycobacterial physiology are modulated by phagosomal acidity and what are the bacterial sensors of phagosomal pH. In this context, an Fe-S cluster containing putative transcription factor WhiB3 is induced in response to acidic pH in medium, inside macrophages, and in the lungs of infected animals (11, 12). In fact, *whiB3* was the only transcription factor whose expression was found to be pH-responsive inside macrophages. *In vitro* studies have shown that WhiB3 responds to dormancy signals, such as O_2_ and nitric oxide (NO), via its 4Fe-4S cluster (13). However, phenotypic experiments revealed no role of WhiB3 in controlling mycobacterial survival in response to NO or hypoxia (13). Therefore, how WhiB3 regulates mycobacterial persistence remained uncharacterized. Consistent induction of *whiB3* in acidic environments *in vitro* and inside macrophages (12) implicated WhiB3 in regulating adaptation of *M. tuberculosis* in response to phagosomal pH. A fundamentally important question remains yet unanswered. What is the mechanism by which WhiB3 mediates the acid response of *M. tuberculosis* during infection?

In this study, we performed global microarray analysis to identify genes regulated by WhiB3 in response to acid stress. More importantly, we measured the dynamic changes in mycothiol redox potential (*E_MSH_*)^3^ of WT *M. tuberculosis* and *Mtb*Δ*whiB3* in response to acidic pH *in vitro* and inside naive and activated macrophages. Confocal studies and host microarray studies were performed to examine the function of WhiB3 in regulating phagosomal maturation. Last, the physiological importance of *whiB3*-mediated effects on gene expression, redox homeostasis, and phagosomal maturation were investigated by performing survival studies in macrophages and guinea pigs. Our study, for the first time, demonstrates how *M. tuberculosis* recalibrates its redox physiology in response to vacuolar pH during infection and identifies a major role of WhiB3 in responding to acidic stress.

## Experimental Procedures

### 

#### 

##### Bacterial Strains and Growth Conditions

Wild type *M. tuberculosis* H37Rv (WT *M. tuberculosis*), *Mtb*Δ*whiB3*, and *whiB3-comp* strains were cultivated as described (13). *E. coli* cultures were grown in LB medium. When required, culture medium was supplemented with kanamycin (25 μg/ml) or hygromycin (50 μg/ml). For acid stress, the pH of 7H9 broth was adjusted using hydrochloric acid (HCl) and buffered using 100 mm MES. Approximately 1 × 10^7^ cells/ml were exposed to various pH-adjusted media, and survival was monitored at day 0 and day 4 by serially diluting the culture and enumerating colony-forming units (cfu). For carbonyl cyanide *m*-chlorophenylhydrazone (CCCP) treatment, bacteria were treated with 500 μm CCCP for 4 h.

##### Generation of MtbΔwhiB3 and whiB3 Complemented Strains

For constructing *Mtb*Δ*whiB3*, 1-kb left flanking and right flanking regions of *whiB3* (Rv3416) were cloned upstream and downstream of the loxP-*gfp*-hygromycin-loxP cassette in a mycobacterial *sacB*-based suicide vector, pML523 (a kind gift from Michael Neiderwies, University of Alabama at Birmingham). The construct was digested with SpeI and NsiI to release left flanking and right flanking regions of *whiB3* along with the loxP-*gfp*-hygromycin-loxP cassette, blunt-ended, and cloned into EcoRV/PstI-digested pRSF-duet vector (Clontech). The pRSF-*whiB3* clone was pretreated with UV as described previously (14)and electroporated into WT *M. tuberculosis* for allelic exchange. The resulting *Mtb*Δ*whiB3* (Hyg^R^Kan^S^GFP^+^) colonies were screened by antibiotic selection and verified by PCR. To unmark *Mtb*Δ*whiB3* strain, pCRE-ZEO-SacB (a gift from Dr. Amit Pandey, Translational Health Science and Technology Institute, Haryana, India) was electroporated into *Mtb*Δ*whiB3*, and the loxP-*gfp*-hygromycin-loxP cassette was released from the genome. The resulting unmarked *Mtb*Δ*whiB3* strain was confirmed by the absence of antibiotic selection marker. Disruption of *whiB3* was confirmed by quantitative RT-PCR (qRT-PCR).

For constructing the *whiB3-comp* strain, the *whiB3* gene along with its promoter region was amplified from the *M. tuberculosis* genome and inserted into an *E. coli*-mycobacterial shuttle vector, pSD5. The pSD-*whiB3* clone was electroporated into unmarked *Mtb*Δ*whiB3* strain to generate the *whiB3-comp* strain. Expression of *whiB3* in the *whiB3-comp* strain was confirmed by qRT-PCR.

##### Cell Lines

The human monocytic cell line THP-1 and mice RAW 264.7 macrophages were cultivated as described previously (15). RAW 264.7 macrophages were activated using 100 ng/ml IFN-γ and 20 ng/ml LPS.

##### Antibodies and Reagents

Antibodies for mammalian markers (CD63 and vacuolar H^+^-ATPase (V-ATPase)) were purchased from Santa Cruz Biotechnology. Lysotracker® DND-red99 was purchased from Thermo Fisher Scientific. Secondary antibody Alexa Fluor 568 was purchased from Thermo Fisher Scientific. Fluorescein 5(6)-isothiocyanate (FITC) was purchased from Sigma-Aldrich. Bafilomycin A1 was purchased from Invivogen, and stocks were made in dimethyl sulfoxide (DMSO), aliquoted, and stored at −20 °C.

##### Growth Conditions for qRT-PCR and Microarray Analysis

For analyzing the influence of acid stress on gene expression, *M. tuberculosis* strains (*M. tuberculosis*, *Mtb*Δ*whiB3*, and *Mtb*Δ*mshA*) were grown until an *A*_600 nm_ of 0.3–0.4 and exposed to 7H9 medium adjusted to a range of acidic pH 4.5, 5.5, 6.2, and 7.0 and normal 7H9 (pH 6.6) for 2 h at 150 rpm at 37 °C. Total RNA from WT *M. tuberculosis* and *Mtb*Δ*whiB3* (pH 4.5 and 6.6) was processed and hybridized to the *M. tuberculosis* whole genome gene expression profiling microarray G2509F (AMADID: G2509F_034585, Agilent Technologies PLC). DNA microarrays were provided by the University of Delhi South Campus MicroArray Centre. RNA amplification, cDNA labeling, microarray hybridization, scanning, and data analysis were performed at the University of Delhi South Campus MicroArray Centre as described (16). Slides were scanned on a microarray scanner (Agilent Technologies) and analyzed using GeneSpring software. Results were analyzed in MeV with significance analysis of microarrays considered significant at *p* ≤ 0.05. The normalized data from the microarray gene expression experiment have been submitted to the NCBI Gene Expression Omnibus and can be queried via Gene Expression Omnibus series accession number GSE61579.

##### qRT-PCR

First-strand cDNA synthesis was performed using 500 ng of the total RNA with the iScript Select cDNA synthesis kit (Bio-Rad) using random oligonucleotide primers. PCR was performed using gene-specific primers (Table 1). Gene expression was analyzed with real-time PCR using iQTM SYBR Green Supermix (Bio-Rad) and a CFX96 RT-PCR system (Bio-Rad). Data analysis was performed with CFX Manager^TM^ software (Bio-Rad). For comparison between WT *M. tuberculosis*, *Mtb*Δ*whiB3*, and the *whiB3-comp* strains, the induction ratio for each gene was normalized to WT *M. tuberculosis* 16S rRNA expression.

**TABLE 1:** **Oligonucleotides used in the study**

Target	Primer sequence
*whiB3* RT F	5′-AACGCAGACATCTGGAACTG-3′
*whiB3* RT R	5′-TAGGGCTCACCGACCTCTAA-3′
*pks2* RT F	5′-AAGTGTCTCCGAGGTGTATG-3′
*pks2* RT R	5′-CGAGTGAAGTGCAGATTACG-3′
*pks3* RT F	5′-GGCTGAGATTGACACTGAAC-3′
*pks3* RT R	5′-TACCCGACATTCCATACGAG-3′
*papA1* RT F	5′-ATCCGCTAAGTACGATGGTC-3′
*papA1* RT R	5′-ACCGATCTAATTGCCCTCAG-3′
Rv3616c RT F	5′-GAGCAGAGCGTTCATCATCG-3′
Rv3616c RTR	5′-CGAACCTAACCAGCCATCAC-3′
Rv2390c RT F	5′-CCGGCGAGTTCAAAGATAAG-3′
Rv2390c RT R	5′-TGAACATCAGGACCACTACC-3′
16S rRNA RT F	5′-GCCGTAAACGGTGGGTACTA-3′
16S rRNA RT R	5′-TGCATGTCAAACCCAGGTAA-3′
*Mtb*Δ*whiB3* clone check F1	5′-GTGGCATCGAGAGCCTCTTCAC-3′
*Mtb*Δ*whiB3* clone check R1	5′-GACGCGTTGATCCTGCTGCAC-3′

##### Measurement of Intramycobacterial E_MSH_ in Vitro and during Infection

For intramycobacterial *E_MSH_* determination, various strains expressing Mrx1-roGFP2 were cultured and exposed to pH stress as indicated above. At the indicated time points, cells were treated with 10 mm *N*-ethylmaleimide for 5 min at room temperature and fixed with 4% paraformaldehyde for 15 min at room temperature. After washing three times with 1× phosphate-buffered saline (PBS), bacilli were analyzed using a FACS Verse Flow cytometer (BD Biosciences). The biosensor response was measured by analyzing the ratio at a fixed emission (510 nm) after excitation at 405 and 488 nm. Data were analyzed using FACSuite software. Intramycobacterial *E_MSH_* was measured using the Nernst equation as described earlier (15).

For measuring intramycobacterial *E_MSH_* during infection, PMA-differentiated THP-1 cells were infected with WT *M. tuberculosis*, *Mtb*Δ*whiB3*, and *whiB3-comp* strains expressing Mrx1-roGFP2 at a multiplicity of infection (MOI) of 10. Infected macrophages were treated with *N*-ethylmaleimide/paraformaldehyde, washed with 1× PBS, and analyzed by flow cytometry as described previously (15). In the case of bafilomycin A1 (BafA1) treatment, THP-1 cells were treated with 10 nm BafA1 (Invivogen) or DMSO (vehicle control) 1 h before infection and processed for infection and sample preparation as described above.

##### Survival of M. tuberculosis Strains in Macrophages

PMA-differentiated THP-1 monocytes and IFNγ + LPS-activated RAW 264.7 macrophages were infected with WT *M. tuberculosis*, *Mtb*Δ*whiB3*, and *whiB3-comp* strains at an MOI of 2 for 4 h, followed by treatment with 200 μg/ml amikacin to remove extracellular bacteria. For BafA1 experiments, THP-1 cells were treated with 10 nm BafA1 or DMSO (vehicle control) 1 h prior to infection. Cells were maintained in BafA1 throughout the experiment. After infection, cells were washed thoroughly with warm RPMI medium and resuspended in 10% RPMI medium containing BafA1 or DMSO as per requirements. Samples were collected at the indicated time points, lysed using 0.06% SDS-7H9, serially diluted in 7H9, plated on OADC-7H11 agar medium, and incubated at 37 °C incubator. Colonies were counted 3 weeks after plating.

##### Isolation of WT M. tuberculosis Surface-exposed Polyketide Lipids

50 ml of WT *M. tuberculosis* was grown in a 37 °C shaker incubator until mid-log phase (*A*_600 nm_ = 0.5–0.8). Surface-exposed extractable total lipids were isolated as described previously (17). For lipid complementation assays, 100 μg/ml lipids were coated on the coverslips, and THP-1 cells were seeded, PMA-differentiated, and infected with *Mtb*Δ*whiB3* as done above.

##### Confocal Microscopy

Logarithmically grown *M. tuberculosis* strains were stained with FITC as described earlier (18). PMA-differentiated THP-1 cells (0.25 × 10^6^) were seeded, and infection was done with WT *M. tuberculosis*, *Mtb*Δ*whiB3*, *whiB3-comp* strains at an MOI of 10 as described elsewhere (19). 1 h prior to the time point, the medium of the cells was replaced with complete medium containing 100 nm Lysotracker® Red DND-99. Staining for CD63 and V-ATPase was started with fixative treatment (4% paraformaldehyde in 1× PBS) for 15 min, followed by permeabilization (0.2% Triton X-100 in 1× PBS) and blocking (3% BSA and 0.5% Tween 80 in 1× PBS). Samples were stained with primary antibodies (anti-CD63 and anti-V-ATPase) followed by secondary antibodies, followed by mounting using ProLong® antifade reagent (Thermo Fisher Scientific). The stained cells were visualized under a Leica TCS SP5 confocal microscope. *z* stacks were taken, collapsed into a two-dimensional image, and analyzed by LAS AF version 2.6.0, build 7266. All of the *z* stacks were taken at 63× oil immersion objective (with zoom). A minimum of five fields were captured, and at least 50 macrophages were analyzed for all of the phagosomal markers, accounting for an approximate analysis of ∼100–200 bacteria/well. For each experimental group, a minimum of three replicates were scored.

##### THP-1 Microarrays

The infection of THP-1 monocytic cells, followed by RNA isolation, sample processing, and hybridization (Illumina Human-Ht-12 BeadChip), was performed as described earlier (19). Array data processing was performed on Illumina Bead Studio software. Expression analysis was done using a volcano plot-based approach using -fold change >1.5 and *p* value >0.05 as cut-off. GO-Elite (version 1.2.5) was used for significant biological analysis of differentially expressed genes with z-score > 1.96 and *p* value < 0.05 set as cut-offs. The normalized data from the microarray gene expression experiment can be queried via Gene Expression Omnibus series accession number GSE65714.

##### Aerosol Infection of Guinea Pigs

Outbred Hartley guinea pigs (∼300–400-g body weight) were given a low dose of logarithmic phase-grown cultures of WT *M. tuberculosis*, *Mtb*Δ*whiB3*, and *whiB3-comp*, using a Madison chamber aerosol generation instrument calibrated to deliver 50–100 cfu/animal lung. Animals were sacrificed (*n* = 5) at 1, 30, and 60 days postinfection for determination of organ bacterial burden and histopathology analysis. Histopathology analysis was performed as described previously (14). A blinded examination of at least three serial sections from each guinea pig was carried out.

##### Statistical Analysis

Statistical analyses were conducted using GraphPad Prism software, and values were presented as mean ± S.D. The statistical significance of the differences between experimental groups was determined by two-tailed, unpaired Student's *t* test. Differences with a *p* value of <0.05 were considered significant.

## Results

### 

#### 

##### M. tuberculosis WhiB3 Regulates Survival and Gene Expression in Response to Acidic pH

Recent studies indicated that intraphagosomal pH might be one of the earliest cues to which *M. tuberculosis* responds and realigns its transcriptional programming (12). Microarray studies have revealed that the expression of *whiB3* was induced early in macrophages in a pH-dependent manner (12). We also found an ∼2-, 4-, and 35-fold increased expression of *whiB3* as compared with 16S rRNA at pH 6.2, 5.5, and 4.5, respectively.

**FIGURE 1.**
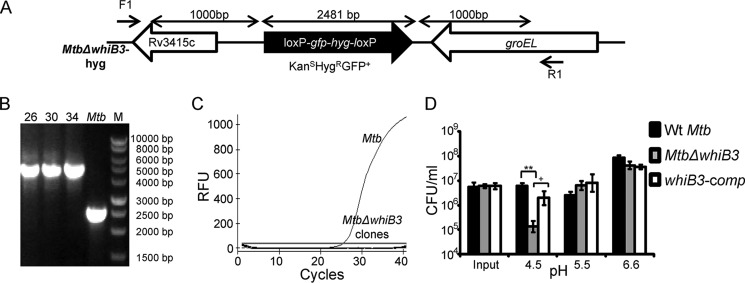
**WhiB3 modulates survival of *M. tuberculosis* at acidic pH.** *A*, schematic representation of disrupted *whiB3* allele (*Rv 3416*) in the genome of *M. tuberculosis*. The entire *whiB3* ORF was replaced by 1-kb right and 1-kb left flanking regions of *whiB3* along with the loxP-*hyg-gfp*-loxP cassette. *B*, genomic DNA was isolated from putative Kan^S^Hyg^R^GFP^+^ *Mtb*Δ*whiB3* colonies (*26*, *30*, and *34*), and replacement of *whiB3* allele with Hyg-GFP cassette was confirmed using PCR with F1 and R1 primers (Table 1). An increase in amplicon size from 2.4 to 4.5 kb due to insertion of the loxP-*hyg-gfp*-loxP cassette was observed in case of mutant clones, confirming the double crossover event. *C*, RNA was isolated from logarithmically grown WT *M. tuberculosis* and the putative *Mtb*Δ*whiB3* clones. qRT-PCR for *whiB3* was done using *whiB3*-specific oligonucleotides (Table 1), and *C_t_* values were plotted to assess the expression. *D*, for assessing survival of *M. tuberculosis* strains at acidic pH, WT *M. tuberculosis*, *Mtb*Δ*whiB3*, and *whiB3-comp* strains were grown in 7H9-tyloxapol medium at the indicated pH values for 4 days at 37 °C, and survival was assessed by enumerating cfu. Data shown are the average of two independent experiments done in triplicate. *Error bars*, S.D. **, *p* < 0.01 (as compared with WT *M. tuberculosis*); +, *p* < 0.05 (as compared with *whiB3-comp*). *RFU*, relative fluorescence units.

Given the sustained induction of *whiB3* in response to acidic pH, we reasoned that WhiB3 might play an important role in tolerating acid stress. We generated an unmarked strain of *M. tuberculosis* lacking the entire open reading frame (ORF) of *whiB3*, and putative *Mtb*Δ*whiB3* clones were verified by PCR (Fig. 1, *A–C*). We monitored the survival of WT *M. tuberculosis*, *Mtb*Δ*whiB3*, and *whiB3-comp* in 7H9-tyloxapol medium adjusted to pH 6.6 (normal 7H9), 5.5, and 4.5. At day 4 post-treatment, survival at various pH stress conditions was monitored by enumerating cfu. We observed that *Mtb*Δ*whiB3* survived to a level comparable with WT *M. tuberculosis* at pH 6.6 and 5.5 (Fig. 1*D*). However, it displayed an ∼55-fold reduction in its survival at pH 4.5 as compared with WT *M. tuberculosis* (*p* = 0.007; Fig. 1*D*). Stable expression of *whiB3* in *Mtb*Δ*whiB3* resulted in a significant complementation of this survival defect (Fig. 1*D*). These observations confirmed that not only is *whiB3* expression induced by acidic pH; it is also required for growth in acidic environments *in vitro*.

**FIGURE 2.**
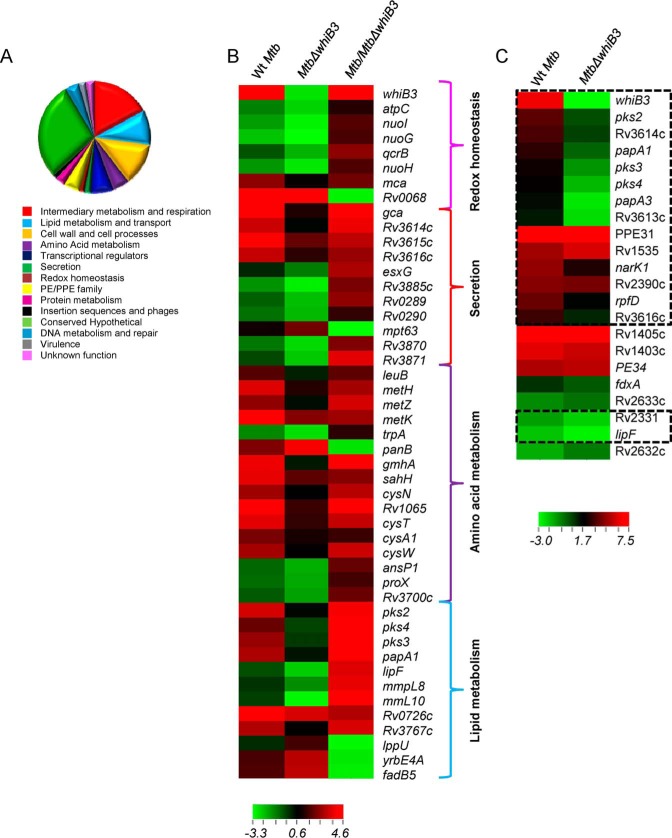
**WhiB3 regulates gene expression in response to acidic pH *in vitro*.** WT *M. tuberculosis* and *Mtb*Δ*whiB3* strains were grown to an *A*_600 nm_ of ∼0.3 and exposed to acidic pH of 4.5 for 2 h at 37 °C. Total RNA was isolated and subjected to microarray analysis as described under “Experimental Procedures.” *A*, genes with >2-fold (*p* < 0.05) up- or down- regulation by acid stress relative to neutral pH were classified in 14 classes based on the annotation given in TubercuList. The *pie chart* represents the relative fraction of various pathways affected by acid stress in WT *M. tuberculosis. B*, heat map comparing WT *M. tuberculosis* and *Mtb*Δ*whiB3* genes induced or repressed significantly (>2-fold, *p* < 0.05) at pH 4.5 relative to neutral pH. *C*, heat map showing the overlap of genes differentially regulated in WT *M. tuberculosis* and *Mtb*Δ*whiB3* by *in vitro* acid stress (pH 4.5) with concanamycin A-sensitive phagosome-induced genes (12). *Highlighted areas* represent genes affected by phagosomal acidification in WT *M. tuberculosis* in a WhiB3-dependent manner at pH 4.5 *in vitro* (1.5-fold up- and down-regulated, *p* < 0.05).

Next, we sought to determine the role of WhiB3 in controlling pH-specific gene expression. We minimized any influence of pH-induced cell death on gene expression by performing microarrays at an early time point (2 h) after exposure to pH 4.5. Expression data revealed differential regulation of several genes involved in secretion, central metabolism/oxidative phosphorylation, lipid metabolism, amino acid metabolism, cell wall biosynthesis/membrane transporters, and gene regulation at pH 4.5 as compared with pH 6.6 (Fig. 2, *A* and *B*, and supplemental Table S1).

Surprisingly, genes directly implicated in mitigating redox stress in *M. tuberculosis* were also influenced by acid stress. For example, expression of major antioxidant genes, including thioredoxins (*trxB1*, *trxB2*, and *trxC*), superoxide dismutase (*sodA*), MSH synthesis (*mshB* and *mca*), and rubredoxin (*rubA* and *rubB*), was elevated by acidic conditions (supplemental Table S1). Genes associated with biosynthesis of redox-active amino acids (cysteine and methionine), DNA repair (*recR*, *dnaK*, *dnaJ*, and *ogt*), and NAD^+^/NADH balance (*ndh*) were also up-regulated (Fig. 2*B* and supplemental Table S1). Noticeably, the expression of *whiB3* was induced to a higher degree than other transcriptional regulators (supplemental Table S1). To understand the physiological basis of our findings, we performed a comparative gene expression analysis at pH 4.5 *in vitro* with the transcriptional changes in WT *M. tuberculosis* in response to early phagosomal acidity (12). We observed that of 22 genes that were induced by early phagosomal acidity (12), 17 were also induced by pH 4.5 (≥2-fold, *p* < 0.05) (Fig. 2*C*).

Subsequently, we examined the role of WhiB3 in regulating pH-induced changes in gene expression. In *Mtb*Δ*whiB3*, the expression of 70 pH-induced and 27 pH-repressed genes showed 2-fold (*p* < 0.05) differential expression as compared with WT *M. tuberculosis* (supplemental Table S1). Several genes exhibited pH-specific induction only in WT *M. tuberculosis*, whereas they were constitutively expressed in *Mtb*Δ*whiB3*. This includes genes involved in the biosynthesis of complex polyketide lipids (sulfolipid-1 (*pks2*), polyacyltrehalose/diacyltrehalose (*pks3-pks4*)), and cysteine metabolism (*cysW*, *cysN*, *cysA1*, and *metZ*) (Fig. 2*B* and supplemental Table S1). Genes involved in amino acid biosynthesis (*metH*, *metK*, and *sahH*), ESX-1 secretion (Rv3614c-Rv3616c), MSH antioxidant system (*mca* and *mtr*), nitrite transport (*narK1*), and leucine biosynthesis (*leuB*) were up-regulated significantly more in WT *M. tuberculosis* as compared with *Mtb*Δ*whiB3* at pH 4.5 (Fig. 2*B* and supplemental Table S1). A large subset of genes was up-regulated to a higher degree in *Mtb*Δ*whiB3* as compared with WT *M. tuberculosis* at acidic pH, indicating the role of WhiB3 in fine tuning the expression of pH-inducible genes. This includes the PE-PPE family (*PE-24*, *PE-8*, *PE-32*, *PPE-65*, and *PPE-31*), ribosomal proteins (*rpmH*, *rplU*, and *rplY*), and transcriptional regulators (*whiB7*, Rv0827c, and Rv3183) (supplemental Table S1). Because induction of *whiB3* was responsive to early phagosomal acidity (12), we checked the expression status of other phagosomal pH-responsive genes in *Mtb*Δ*whiB3*. We discovered that of 22 phagosomal pH-responsive genes, expression of 16 was controlled by WhiB3 (Fig. 2*C*, ≥1.5-fold, *p* ≤0.05). Last, microarray data were validated by measuring the expression of a selected set of pH- and *whiB3*-dependent genes by qRT-PCR (Table 2). Taken together, our results implicate WhiB3 in controlling the survival and expression of genes involved in altering cell wall lipid composition, secretion, and redox balance in response to acid stress.

**TABLE 2:** **qRT-PCR analysis of a select set of pH-specific genes regulated by WhiB3 and mycothiol under acidic stress (pH 4.5)** Expression is compared within various treatment groups, and data are represented as -fold change in gene expression ± S.D. ND, not determined.

Gene	WT *M. tuberculosis* pH 4.5/WT *M. tuberculosis* pH 6.6	*Mtb*Δ*whiB3* pH 4.5/WT *Mtb* pH 6.6	WT *M. tuberculosis* pH 4.5/*Mtb*Δ*whiB3* pH 4.5	WT *M. tuberculosis* pH 7.0/*Mtb*Δ*mshA* pH 7.0	*Mtb*Δ*mshA* pH 4.5/*Mtb*Δ*mshA* pH 7.0
*whiB3*	26 ± 1.6	ND	ND	−1.1 ± 0.47	−37.63 ± 13.11
*pks2*	12 ± 0.8	1.5 ± 0.24	2 ± 0.31	−1.66 ± 0.805	−4.9 ± 1.91
*pks3*	5.17 ± 2.8	0.55±.0.16	9.3 ± 0.62	−1.15 ± 0.17	−5.11 ± 2.46
*papA1*	4.6 ± 2.25	0.71 ± 0.07	6.1 ± 0.53	−0.2 ± 1.93	−12.43 ± 4.76
Rv3616c	14.8 ± 4.1	2.3 ± 1	8 ± 0.52	−1.46 ± 2.48	1.96 ± 0.26
Rv2390c	54 ± 26	14.6 ± 3.1	3.7 ± 0.53	1.19 ± 0.36	3.03 ± 0.75

##### Acidic pH Induces Dynamic Changes in E_MSH_ of M. tuberculosis in a WhiB3-dependent Manner

Because WhiB3 is believed to serve as an intracellular redox sensor in *M. tuberculosis*, we hypothesized that it might regulate gene expression by responding to pH-induced changes in intramycobacterial redox physiology. Therefore, we precisely measured the dynamic changes in *E_MSH_* of WT *M. tuberculosis*, *Mtb*Δ*whiB3*, and *whiB3-comp* strains in response to a range of pH conditions*in vitro*. We exploited a highly sensitive, specific, and non-invasive biosensor of *E_MSH_* (Mrx1-roGFP2) in mycobacteria (15). MSH is the most abundant low molecular weight thiol produced by mycobacteria (15). Therefore, *E_MSH_* measurement provides a reliable and sensitive numerical evaluation of cytoplasmic redox state of mycobacteria. The biosensor shows an increase in the fluorescence excitation ratio at 405/488 nm upon oxidative stress, whereas a ratiometric decrease is associated with reductive stress (15).

**FIGURE 3.**
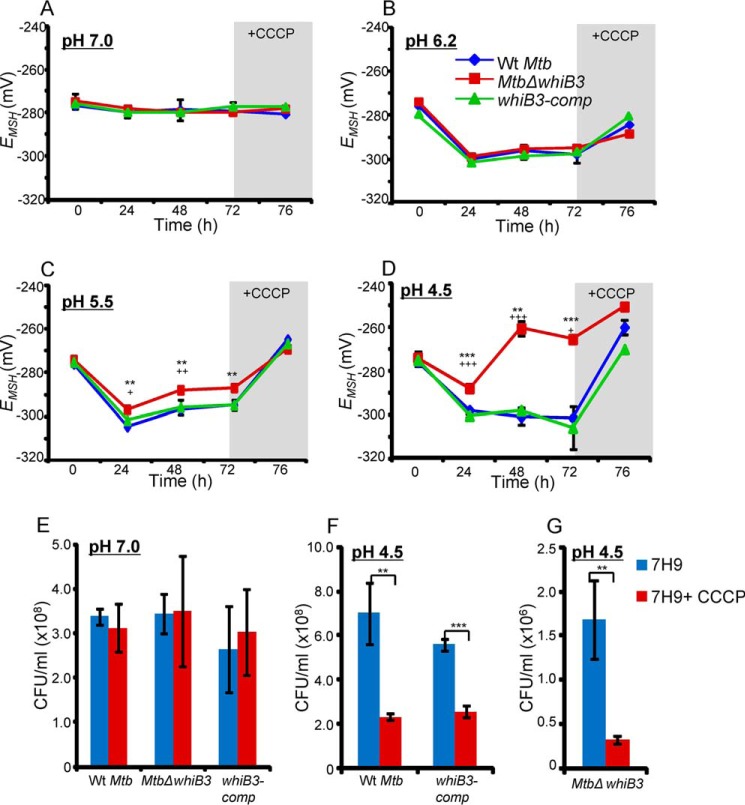
**WhiB3 regulates dynamic changes in intramycobacterial *E_MSH_* of *M. tuberculosis* in response to acid stress.** WT *M. tuberculosis*, *Mtb*Δ*whiB3*, and *whiB3-comp* strains expressing Mrx1-roGFP2 were grown in 7H9-tyloxapol medium at a pH of 7.0 (*A*), 6.2 (*B*), 5.5 (*C*), and 4.5 (*D*). At the indicated time points, *E_MSH_* of 30,000 bacterial cells was measured by flow cytometry as described under “Experimental Procedures.” At 72 h postincubation, 500 μm CCCP was added to dissipate the pH gradient, and intramycobacterial *E_MSH_* was measured 4 h post-CCCP treatment (*i.e.* 76 h). **, *p* < 0.01; ***, *p* < 0.005 (as compared with WT *M. tuberculosis*); +, *p* < 0.05; ++, *p* < 0.01; +++, *p* < 0.005 (as compared with *whiB3-comp*). Shown is the viability of *WT M. tuberculosis*, *Mtb*Δ*whiB3*, and *whiB3-comp* upon CCCP treatment at pH 7.0 (*E*) or pH 4.5 (*F* and *G*) for 4 h, as determined by enumerating cfu. Data shown in each *panel* are the result of at least two independent experiments performed in triplicate. *Error bars*, S.D. **, *p* < 0.01; ***, *p* < 0.005.

WT *M. tuberculosis*, *Mtb*Δ*whiB3*, and *whiB3-comp* expressing Mrx1-roGFP2 were exposed to a gradient of pH conditions (pH 7.0, 6.2, 5.5, and 4.5), and the ratiometric response was measured by flow cytometry at various time points. By fitting ratiometric intensities into the Nernst equation, we precisely measured the *E_MSH_* of *M. tuberculosis* strains at various pH conditions (see “Experimental Procedures”). We found the steady-state *E_MSH_* of WT *M. tuberculosis*, *Mtb*Δ*whiB3*, and *whiB3-comp* strains at neutral pH to be approximately −275 mV (Fig. 3*A*). The comparable *E_MSH_* of *Mtb*Δ*whiB3* and WT *M. tuberculosis* indicates that WhiB3 is not required for maintaining ambient *E_MSH_* of *M. tuberculosis* at neutral pH. Interestingly, 24-h exposure of WT *M. tuberculosis* to either pH 6.2, pH 5.5, or pH 4.5 resulted in a significant decrease in intramycobacterial *E_MSH_* (approximately −305 ± 0.7 mV), indicating that a transition from neutral to either milder or harsher acidic pH conditions uniformly induces reductive *E_MSH_* in *M. tuberculosis* (Fig. 3, *B–D*). Moreover, pH-exposed WT *M. tuberculosis* largely maintained *E_MSH_* reduced throughout the course of the experiment. We noted a very modest recovery from reductive *E_MSH_* (*i.e.* approximately −295 mV) at pH 6.2 and 5.5 at later time points, whereas no such effect was observed at pH 4.5 (Fig. 3, *B–D*). Importantly, whereas changes in intramycobacterial *E_MSH_* for *Mtb*Δ*whiB3* mostly followed the WT *M. tuberculosis* pattern at pH 6.2 (Fig. 3*B*), distinct redox deviations were observed at pH 5.5 and 4.5. For example, at pH 5.5, *Mtb*Δ*whiB3* displayed a relatively lesser decrease in intramycobacterial *E_MSH_* (−297 ± 0.7 mV) at 24 h followed by a slightly better recovery at 48–72 h (approximately −287 ± 0.7 mV) as compared with WT *M. tuberculosis* (Fig. 3*C*). Noticeably, exposure of *Mtb*Δ*whiB3* to pH 4.5 displayed only a marginal decrease in *E_MSH_* (−287 ± 1.5 mV) at 24 h, followed by a significant increase in intramycobacterial *E_MSH_* at 48 h (−260.5 ± 3.5 mV) and at 72 h (−266 ± 0.7 mV) as compared with WT *M. tuberculosis*, suggesting an overall oxidative shift in *E_MSH_* of *Mtb*Δ*whiB3* (Fig. 3*D*). The complemented strain showed changes in intramycobacterial *E_MSH_* that were comparable with WT *M. tuberculosis* (Fig. 3, *A–D*). These results indicate that acidic pH perturbs the redox physiology of *M. tuberculosis* by inducing a reductive shift in intramycobacterial *E_MSH_* and that the loss of WhiB3 impaired the ability of *M. tuberculosis* to orchestrate an efficient and dynamic MSH-specific reductive response upon acid stress.

The above results point toward a pH-mediated reductive shift in *E_MSH_* of *M. tuberculosis* that might act as a signal for WhiB3 to regulate gene expression. We reasoned that disruption of the MSH reductive pathway would provide an ideal opportunity to study, in parallel, the effect of reductive *E_MSH_* on pH-specific gene expression. Therefore, we expressed Mrx1-roGFP2 in an MSH-deficient *M. tuberculosis* strain (*Mtb*Δ*mshA*) and found that *E_MSH_* of the strain remained oxidized (∼−240 mV) at various pH conditions (7.0, 6.2, 5.5, and 4.5). Next, we analyzed the expression of a set of pH-inducible genes in a MSH-deficient *M. tuberculosis* strain (*Mtb*Δ*mshA*) by qRT-PCR. At pH 7.0, expression of pH-inducible genes in *Mtb*Δ*mshA* is comparable with WT *M. tuberculosis* (Table 2). In contrast, expression of pH-inducible genes did not show any up-regulation in *Mtb*Δ*mshA* at pH 4.5 (Table 2). More importantly, the expression of *whiB3* was ∼37-fold down-regulated in *Mtb*Δ*mshA* at pH 4.5 (Table 2). These results, along with our microarray data showing *whiB3*-dependent expression of MSH biosynthetic genes, suggest that MSH and WhiB3 are the components of a regulatory circuit mediating gene expression upon acid stress.

##### Dissipation of pH Gradient Perturbs pH-specific Induction of Reductive E_MSH_ in M. tuberculosis

Excess increase in intracellular acidity can damage DNA, proteins, and lipids to exert bacterial killing. However, whether acid is the main effector of bacterial killing in response to pH stress is not clear. Because *M. tuberculosis* maintains cytoplasmic pH homeostasis even under severe pH stress, how an increase in internal acidity will affect mycobacterial redox physiology and survival has not been studied. To examine the influence of elevated cytoplasmic acidity on intramycobacterial redox potential, we cultured *M. tuberculosis* strains at various pH levels for 72 h, as described above, and disrupted pH homeostasis by discharging the pH gradient (ΔpH) using a well known protonophore, CCCP. Treatment with 500 μm CCCP for 4 h is sufficient to permeate the cell wall of mycobacteria and equilibrates cytoplasmic pH with the external pH (20). We followed intramycobacterial *E_MSH_* and viability of *M. tuberculosis* strains at 4 h post-treatment with 500 μm CCCP. Expectedly, at pH 7.0, treatment with CCCP has no effect on either intramycobacterial *E_MSH_* or viability of *M. tuberculosis* (Fig. 3, *A* and *E*). At pH 6.2, CCCP treatment triggers a moderate recovery from reductive shift in *E_MSH_* of WT *M. tuberculosis* (−284 mV), *Mtb*Δ*whiB3* (−288 mV), and *whiB3-comp* (−280 mV) strains (Fig. 3*B*). However, under these experimental conditions, viability of *M. tuberculosis* strains was not significantly compromised (data not shown).Consistent with this pattern, CCCP treatment of *M. tuberculosis* strains at pH 5.5 and 4.5 completely reversed the pH-specific increase in reductive *E_MSH_*, as indicated by an increased shift in intramycobacterial *E_MSH_* toward oxidizing (ranging from −260 to −250 mV) (Fig. 3, *C* and *D*). Although an increase in oxidative stress upon CCCP treatment does not significantly influence the viability of *M. tuberculosis* strains at pH 5.5 (data not shown), an ∼3–4 fold reduction in cfu was observed at pH 4.5 (Fig. 3, *F* and *G*). Importantly, because a CCCP-mediated skew toward *E_MSH_*-oxidized was activated at moderate pH values of 5.5, at which ionophore showed no mycobactericidal effect, our results indicate that dissipation of ΔpH and consequent oxidative shift in intramycobacterial *E_MSH_* precedes bacterial death. Together, these results suggest that the maintenance of pH homeostasis and the induction of reductive *E_MSH_* are effective and overlapping mycobacterial strategies to avoid oxidative stress-mediated death caused by increased internal acidity.

##### WhiB3 Regulates Intramycobacterial E_MSH_ and Survival during Infection

We next determined whether WhiB3 regulates intramycobacterial *E_MSH_* and survival in the natural context of infection. To investigate this issue, we infected THP-1 macrophages with *M. tuberculosis* strains expressing Mrx1-roGFP2 at an MOI of 10 and monitored intramycobacterial *E_MSH_* and survival, as described previously (15).

**FIGURE 4.**
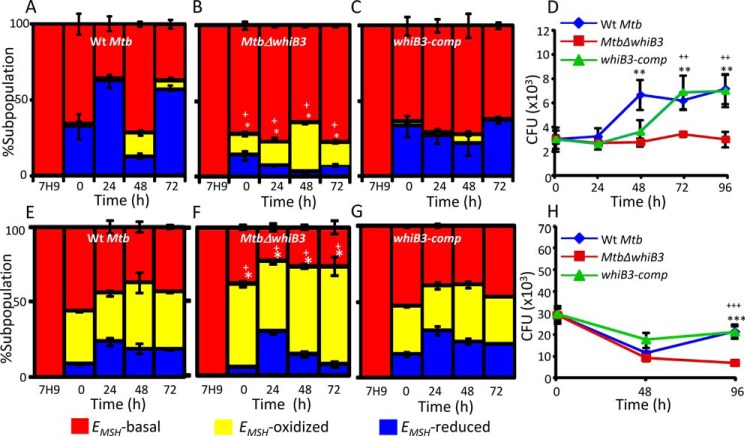
**Regulation of intramycobacterial *E_MSH_* and survival of *M. tuberculosis* in a WhiB3-dependent manner inside macrophages.** THP-1 cells were infected with Mrx1-roGFP2 expressing WT *M. tuberculosis* (*A*), *Mtb*Δ*whiB3* (*B*), and *whiB3-comp* (*C*) strains at an MOI of 10. At the indicated time points, ∼30,000 infected macrophages were analyzed by flow cytometry, intramycobacterial *E_MSH_* was measured, and the percentage of bacilli in each subpopulation was determined as described earlier (15). The percentage of bacilli in each subpopulation (*E_MSH_*-oxidized, *E_MSH_*-basal, and *E_MSH_*-reduced) was plotted as a *bar graph* as described earlier (15). The 0 h time point refers to time immediately after infection with *M. tuberculosis* strains for 6 h (4 h of internalization followed by 2 h of amikacin treatment to remove any extracellular bacilli). *D*, THP-1 macrophages were infected with WT *M. tuberculosis*, *Mtb*Δ*whiB3*, and *whiB3-comp* strains as described above, and intramacrophage survival was monitored by enumerating cfu at the indicated time points. IFN-γ- and LPS-treated (activated) RAW 264.7 macrophages were infected with Mrx1-roGFP2 expressing WT *M. tuberculosis* (*E*), *Mtb*Δ*whiB3* (*F*), and *whiB3-comp* strains (MOI of 10) (*G*). At the indicated time points, intramycobacterial *E_MSH_* of *M. tuberculosis* subpopulations was measured as described for *A–C. H*, activated RAW 264.7 macrophages were infected with WT *M. tuberculosis*, *Mtb*Δ*whiB3*, and *whiB3-comp* strains (MOI of 2), and intramacrophage survival was monitored as described for *D*. The data in *all panels* are representative of three independent experiments performed in quadruplicate. *Error bars*, S.D. *, *p* < 0.05 (*E_MSH_*-oxidized subpopulation of *Mtb*Δ*whiB3* as compared with WT *M. tuberculosis*); +, *p* < 0.05 (*E_MSH_*-oxidized subpopulation of *Mtb*Δ*whiB3* as compared with *whiB3 Comp*) in experiments related to measurements of *E_MSH_*. **, *p* < 0.01; ***, *p* < 0.005 (as compared with WT *M. tuberculosis*); ++, *p* < 0.01; +++, *p* < 0.005 (as compared with *whiB3-comp*) in intramacrophage survival experiments.

As expected, *M. tuberculosis* strains displayed subpopulations with oxidized (−240 ± 3 mV), reduced (−300 ± 6 mV), and basal (−275 ± 5 mV) *E_MSH_* inside THP-1 cells (15). The WT *M. tuberculosis* displayed a gradual increase in population with *E_MSH_* reduced (∼30–65%) at initial time points (0–24 h postinfection), followed by an increase in *E_MSH_*-oxidized population (∼20%) at the intermediate period (48 h postinfection) and recovery from oxidative stress at 72 h postinfection. (Fig. 4*A*). In contrast to what was observed with *whiB3*-sufficient strains (WT *M. tuberculosis* and *whiB3-comp*), *Mtb*Δ*whiB3* showed negligible proportions of *E_MSH_*-reduced bacteria and a greater fraction of bacteria with *E_MSH_* oxidized, at each time point examined (Fig. 4, *A–C*). The defective ability of *Mtb*Δ*whiB3* to maintain reductive *E_MSH_* correlated with a significant growth defect in its survival inside THP-1 macrophages as compared with WT *M. tuberculosis* and *whiB3-comp* (Fig. 4*D*).

Activated murine macrophages are known to control mycobacterial survival by generating excessive acid, ROS, and RNS stress (1). To investigate the role of WhiB3 in controlling *E_MSH_* of *M. tuberculosis* upon stimulation of antimycobacterial stresses, we infected IFN-γ- and LPS-activated RAW264.7 cells with WT *M. tuberculosis*, *Mtb*Δ*whiB3*, and *whiB3-comp* at an MOI of 10 and measured the intramycobacterial *E_MSH_*. In a parallel experiment, intramacrophage survival of *M. tuberculosis* strains was also compared upon immune activation. We observed a substantial shift in subpopulation with *E_MSH_* oxidized in all three strains (Fig. 4, *E–G*). However, *Mtb*Δ*whiB3* displayed the highest proportion of cells with *E_MSH_* oxidized (Fig. 4*F*). Inside immune activated macrophages, WT *M. tuberculosis* showed a decline in survival at 48 h postinfection, followed by a significant recovery at 96 h postinfection (Fig. 4*H*). However, although initial killing of *Mtb*Δ*whiB3* was comparable with WT *M. tuberculosis*, the mutant showed a 2-fold difference in survival at 96 h postinfection (*p* = 0.0018) (Fig. 4*H*). The *whiB3-comp* strain grew equivalent to WT *M. tuberculosis* levels inside activated macrophages (Fig. 4*H*).

An oxidative shift in the *E_MSH_* of WT *M. tuberculosis* inside activated macrophages, where phagolysosomal pH drops to 4.5, is in contrast with the reductive shift in intramycobacterial *E_MSH_* at pH 4.5 in 7H9 culture medium. However, these counterintuitive findings can be reconciled by the synergistic effect of vacuolar acidification with host ROS and RNS production. In particular, low pH of phagolysosomes allows dismutation of nitrous acid (generated via autoxidation of NO) to generate highly toxic nitrogen dioxide (21). Hence, the increase in *E_MSH_*-oxidized subpopulations is most likely due to composite response of *M. tuberculosis* toward multiple stresses encountered inside activated macrophages. Altogether, using *in vitro* and macrophage-based assays, we demonstrated the role of WhiB3 in mounting an efficient antioxidant response to promote intramacrophage survival.

##### Effect of Phagosomal Acidification on Intramycobacterial E_MSH_ and Survival

Because phagosomal pH synergizes with multiple intramacrophage cues, such as ROS and RNS, determination of the specific effect of acidic pH on mycobacterial physiology, gene expression, and survival remains challenging. This has been extremely difficult inside activated macrophages, where several mycobactericidal mechanisms (*e.g.* lysosomal hydrolases, ROS, and RNS) are likely to be pH-dependent (21). Therefore, to begin delineating the role of vacuolar acidification on intramycobacterial *E_MSH_*, we decided to examine the effect of limited acidification encountered inside THP-1 cells (22). To block phagosomal acidification, we treated THP-1 macrophages with a specific inhibitor of V-ATPase, BafA1, and then infected them with *M. tuberculosis* strains expressing Mrx1-roGFP2 at an MOI of 10. Treatment with 10 nm BafA1 is known to effectively block phagosomal acidification without affecting macrophage viability during infection with *M. tuberculosis* (23). We also observed no influence of 10 nm BafA1 on THP-1 viability (data not shown). Given that *M. tuberculosis* phagosomes acidify to pH 6.2–6.4 within minutes of infection (24), we measured intramycobacterial *E_MSH_* at initial time points (0 and 24 h postinfection).

**FIGURE 5.**
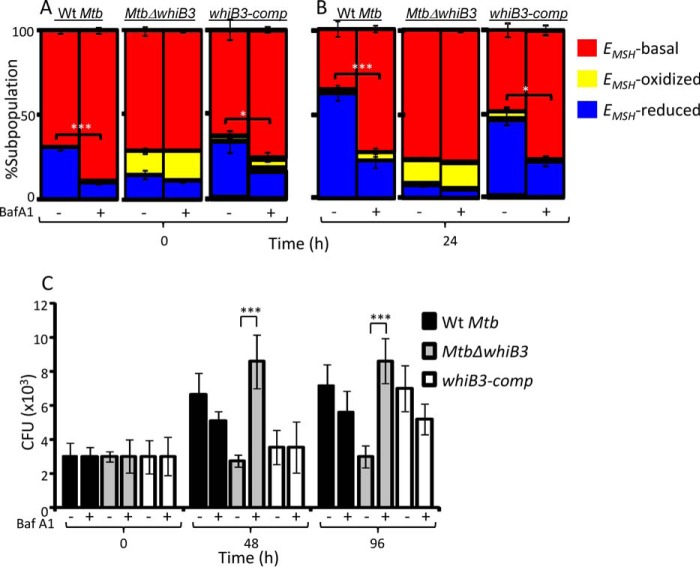
**Phagosomal acidification modulates *E_MSH_* and survival of *M. tuberculosis* in a WhiB3-dependent manner.** *A* and *B*, THP-1 cells treated with 10 nm BafA1 or vehicle control (DMSO) were infected with WT *M. tuberculosis*, *Mtb*Δ*whiB3*, and *whiB3-*c*omp* strains (MOI of 10), and at the indicated time points, 30,000 infected cells were analyzed by flow cytometry, and relative distribution of *M. tuberculosis* subpopulations with varying intramycobacterial *E_MSH_* was measured as described earlier (15). The data are represented as a percentage of bacilli in each subpopulation ± S.D. *, *p* < 0.05; ***, *p* < 0.005 (*E_MSH_*-reduced subpopulation in BafA1-untreated as compared with BafA1-treated cells). *C*, THP-1 cells treated with BafA1 or vehicle control (DMSO) were infected with WT *M. tuberculosis*, *Mtb*Δ*whiB3*, and *whiB3-comp* strains, and intramacrophage survival at the indicated time points was monitored by enumerating cfu. The data in *all panels* are representative of three independent experiments performed in quadruplicate. *Error bars*, S.D. ***, *p* < 0.005 (BafA1-treated *Mtb*Δ*whiB3-*infected THP-1 macrophages as compared with untreated control).

In case of WT *M. tuberculosis*, BafA1 treatment significantly diminished the proportion of bacilli with *E_MSH_* reduced as compared with untreated macrophages at 0 and 24 h postinfection (Fig. 5, *A* and *B*). The consequent increase in *M. tuberculosis* subpopulations with basal *E_MSH_* (similar to 7H9, pH 7.0, grown bacteria) indicates that cells are experiencing nearly neutral pH in vacuoles upon BafA1 treatment (Fig. 5, *A* and *B*). Importantly, these results indicate that limited acidification encountered inside macrophages is sufficient to induce reductive shift and heterogeneity in *E_MSH_* of *M. tuberculosis* during infection. In contrast to WT *M. tuberculosis*, *Mtb*Δ*whiB3* showed no significant changes in subpopulations with basal, oxidized, and reduced *E_MSH_* at 0 and 24 h postinfection upon BafA1 treatment (Fig. 5, *A* and *B*). The *whiB3*-c*omp* strain showed intramycobacterial *E_MSH_* changes comparable with WT *M. tuberculosis* upon BafA1 treatment (Fig. 5, *A* and *B*). These results suggest that the lack of WhiB3 impaired the ability of *M. tuberculosis* to dynamically modulate cytoplasmic *E_MSH_* in response to vacuolar pH.

Next, we examined whether the inability of *Mtb*Δ*whiB3* to maintain mycothiol redox homeostasis in response to vacuolar acidification was the reason underlying the intramacrophage survival defect of the mutant. To do this, we monitored the survival of WT *M. tuberculosis*, *Mtb*Δ*whiB3*, and *whiB3-comp* strains inside untreated and BafA1-treated THP-1 cells by enumerating cfu at 48 and 96 h postinfection. In contrast to defective intramacrophage growth of *Mtb*Δ*whiB3* observed earlier, treatment with BafA1 completely rescued its survival to WT *M. tuberculosis* and *whiB3-comp* levels (Fig. 5*C*). Together, these results indicate the importance of mycothiol redox buffer and WhiB3 in responding to phagosomal acidification and maintaining intramacrophage survival of *M. tuberculosis*.

##### WhiB3 Is Required to Subvert Phagosomal Acidification

One of the main mechanisms exploited by *M. tuberculosis* to resist acid stress is by blocking the normal process of phagosomal maturation to acidified phagolysosomes (4). In this regard, we have shown that the majority of bacilli within acidified phagolysosomal fractions display oxidative *E_MSH_* (15). Because a relatively higher proportion of *E_MSH_*-oxidized subpopulations of *Mtb*Δ*whiB3* were detected inside THP-1 cells, we hypothesized that increased fusion of phagosomes containing *Mtb*Δ*whiB3* with acidified lysosomes may be one of the factors that underlies the observed redox variability and intramacrophage survival defect. We therefore sought to determine the acidification status of phagosomes containing *Mtb*Δ*whiB3*. To do this, THP-1 macrophages were infected with *M. tuberculosis* strains labeled with FITC at an MOI of 10, and localization of *M. tuberculosis* bacilli was assessed using well established markers of phagosomal maturation. Localization of *M. tuberculosis* bacilli was examined using confocal microscopy. For measurements, a minimum of five fields/well were captured, and ∼100–200 bacterium-containing phagosomes were scored per well. For each test group, three replicate wells were scored per experiment.

**FIGURE 6.**
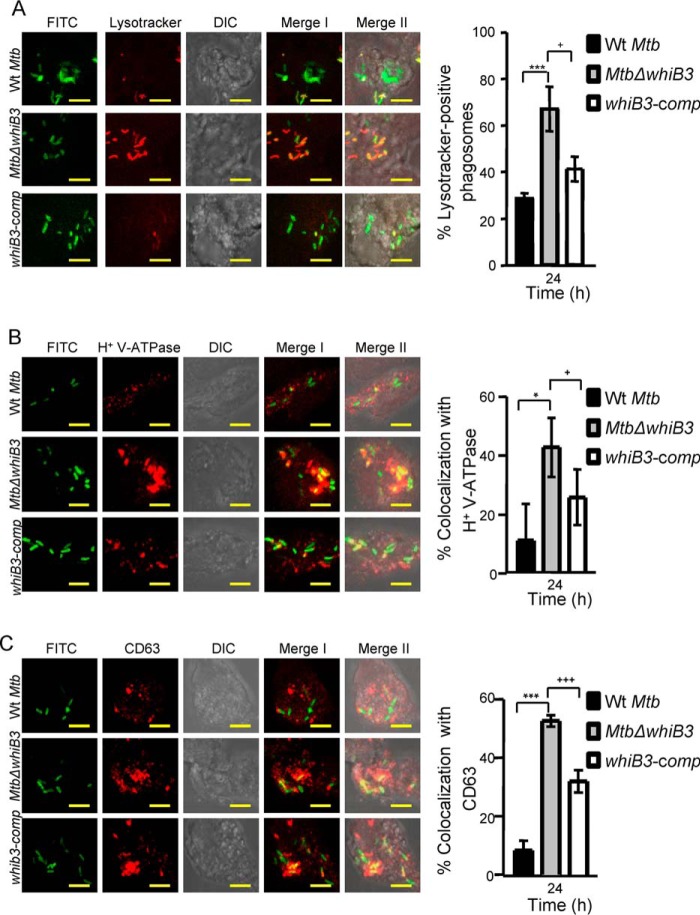
***Mtb*Δ*whiB3* is enriched in phagosomes positive for late endosomal/lysosomal markers inside THP-1 macrophages.** THP-1 cells were infected with FITC-labeled WT *M. tuberculosis*, *Mtb*Δ*whiB3*, and *whiB3-comp* strains (MOI of 10). At 24 h postinfection, cells were independently stained with Lysotracker (*A*) or immunofluorescent antibodies for markers of phagosome maturation (*i.e.* V-ATPase (*B*) and CD63 (*C*), as described under “Experimental Procedures”). A representative result of three independent experiments is shown. In the images, *green* indicates FITC-labeled bacteria; *red* indicates Lysotracker or V-ATPase or CD63; differential interference contrast (*DIC*) indicates cell morphology; and *yellow* indicates merged images of two signals (*Merge I*, FITC/phagosomal markers) and three signals (*Merge II*, FITC/phagosomal markers/DIC). *Scale bar*, 5 μm. The *bar graphs* represent mean percentages of bacterium-containing phagosomes that stain positive for markers. *Error bars*, S.D. of three replicate wells, with each well having ∼100–200 phagosomes scored. *, *p* < 0.05; ***, *p* < 0.005 (as compared with WT *M. tuberculosis*); +, *p* < 0.05; +++, *p* < 0.005 (as compared with *whiB3-comp*).

Staining with Lysotracker revealed that WT *M. tuberculosis* largely remained in non-acidified phagosomes, with only 25 ± 4% of bacilli colocalized to acidified phagosomes at 24 h postinfection (Fig. 6*A*). In contrast, a significantly greater fraction of *Mtb*Δ*whiB3* (∼70%, *p* = 0.0026) was found in acidified phagosomes, and this phenotype was significantly reversed in the complemented strain (Fig. 6*A*). It has been shown that *M. tuberculosis* actively inhibits phagosome acidification by preventing recruitment and/or inducing degradation of a molecular proton motor, V-ATPase (24, 25). Therefore, we questioned whether the increased association of *Mtb*Δ*whiB3* with acidified phagosomes correlates with greater V-ATPase association. Infected THP-1 macrophages were immunostained for human V-ATPase, and colocalization was measured at 24 h postinfection. A significantly greater percentage of phagosomes containing *Mtb*Δ*whiB3* were found to be positive for V-ATPase (∼43 ± 10%) as compared with WT *M. tuberculosis* (∼11 ± 12%) or *whiB3-comp* (∼25 ± 10%) (Fig. 6*B*). Because phagosome acidification is a relatively early step in phagosomal maturation, we next analyzed the status of *Mtb*Δ*whiB3-*containing phagosomes for the late endosome-lysosome fusion marker, CD63. It has been reported that WT *M. tuberculosis* prevents phagosomes from maturing into the CD63-positive state (26). Consistent with our earlier results, ∼53 ± 2% of *Mtb*Δ*whiB3-*containing phagosomes acquired CD63 as compared with ∼8 ± 3% and 31 ± 4% in the case of WT *M. tuberculosis* and *whiB3-comp* strains, respectively (Fig. 6*C*). The observed differences in the phagosomes of *Mtb*Δ*whiB3* were confirmed by repeating experiments at least three times in triplicate.

**FIGURE 7.**
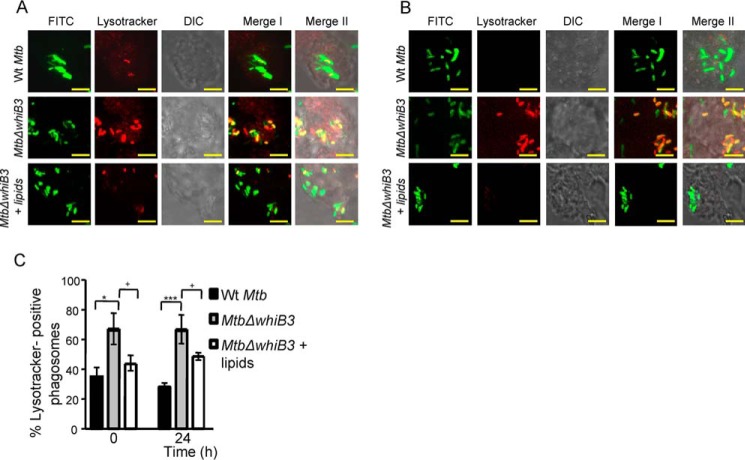
**Treatment with surface-exposed lipids derived from WT *M. tuberculosis* reduced the enrichment of *Mtb*Δ*whiB3* in acidified phagosomes**. Total surface-exposed lipids were extracted from WT *M. tuberculosis* and coated onto coverslips, followed by seeding of THP-1 cells as described under “Experimental Procedures.” Lipid- pretreated macrophages were infected with FITC-labeled *Mtb*Δ*whiB3* (MOI of 10). As a control, untreated THP-1 cells were infected with FITC-labeled WT *M. tuberculosis* and *Mtb*Δ*whiB3* (MOI of 10). At 0 h (*A*) and 24 h (*B*) postinfection, cells were stained with Lysotracker and analyzed by confocal microscopy. Representative microscopy images from at least three independent experiments for each time point are shown. In the images, *green* indicates FITC-labeled bacteria, *red* indicates Lysotracker, differential interference contrast (DIC) indicates cell morphology, and *yellow* indicates merged images of two signals (*Merge I*, FITC/phagosomal markers) and three signals (*Merge II*, FITC/phagosomal markers/DIC). *Scale bar*, 5 μm. *C*, mean percentages of bacterium-containing phagosomes that stain positive for Lysotracker. *Error bars*, S.D. of three replicate wells, with each well having >100 phagosomes scored. *, *p* < 0.05; ***, *p* < 0.005 (as compared with WT *M. tuberculosis*); +, *p* < 0.05; ++, *p* < 0.01 (as compared with *whiB3-comp*).

Our data show that WhiB3 positively regulates the pH-specific expression of various genes involved in producing secretory proteins and lipids (*e.g.* sulfolipid-1 (*pks2*, *papA1*, and *mmpL8*), TDM (*fabD*, *acpP*, and *kasA*), and ESX-1 system (Rv3615c, Rv3870, and Rv3871)), which are well known to restrict phagosomal maturation (27–29). Hence, the inability of *Mtb*Δ*whiB3* to block phagosomal maturation could be a consequence of defective polyketide surface lipid anabolism. To investigate this possibility, total surface exposed lipids were extracted from WT *M. tuberculosis* as described earlier (17). Moreover, pretreatment of macrophages with the surface-exposed lipids of mycobacteria has been shown to modulate cellular processes, such as phagosomal maturation and autophagy (30, 31). Based on these studies, THP-1 cells were pretreated with the surface lipids and subsequently infected with WT *M. tuberculosis* or *Mtb*Δ*whiB3* at an MOI of 10. Infected macrophages were assessed for the colocalization of Lysotracker with *Mtb*Δ*whiB3* at 0 and 24 h postinfection. Assessment of more than 100 phagosomes revealed that pretreatment with the WT *M. tuberculosis* lipids resulted in a significant inhibition of Lysotracker staining of *Mtb*Δ*whiB3*-containing phagosomes as compared with untreated controls at each time point tested (Fig. 7, *A–C*). Importantly, the percentage of Lysotracker-positive phagosomes containing *Mtb*Δ*whiB3* upon pretreatment with total lipids was comparable with WT *M. tuberculosis* at 0 h postinfection (Fig. 7, *A* and *C*). These results suggest that the altered composition of surface-associated polyketide lipids is likely to be one of the factors responsible for the defective ability of *Mtb*Δ*whiB3* to block phagosomal maturation. In sum, the data generated from macrophages and *in vitro* experiments clearly suggest that WhiB3 protects *M. tuberculosis* from acid stress by regulating gene expression, dynamic changes in *E_MSH_* of *M. tuberculosis*, and polyketide-mediated restriction of phagosomal acidification.

##### WhiB3 Modulates the Expression of Host Innate Response Genes

**FIGURE 8.**
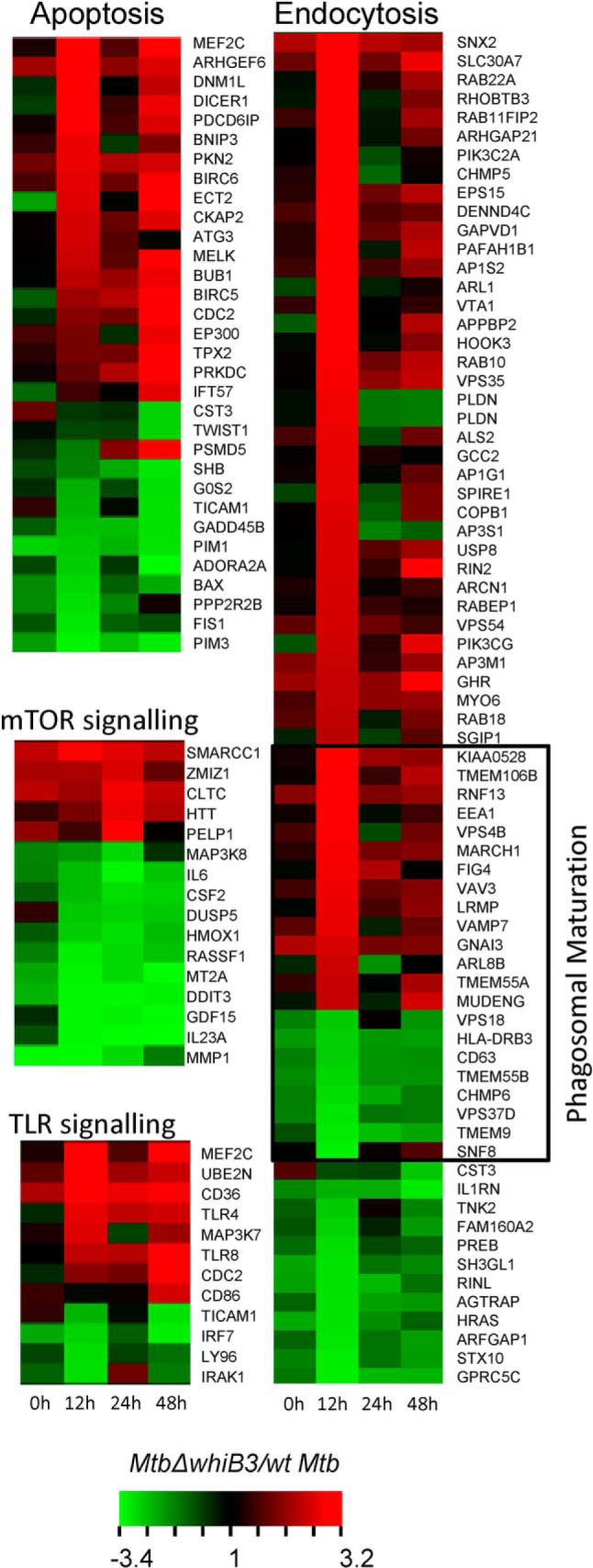
**Regulation of host transcriptome by *M. tuberculosis* WhiB3.** PMA-differentiated THP-1 cells were infected with WT *M. tuberculosis* and *Mtb*Δ*whiB3.* At the indicated time points, total RNA from THP-1 was isolated and transcriptomic analysis was done, as described under “Experimental Procedures.” The figure shows a *heat map* of major pathways differentially regulated in *Mtb*Δ*whiB3* as compared with *M. tuberculosis*, >1.5-fold (*p* < 0.05).

We examined whether WhiB3-mediated regulation of bioactive lipids and secretory proteins modulates expression of host transcriptome. Using microarrays, we compared the expression of THP-1 cells infected with WT *M. tuberculosis* and *Mtb*Δ*whiB3* at various time points. We found that major innate immune mechanisms normally suppressed by pathogenic *M. tuberculosis* strains, such as phagosomal maturation/endocytosis, apoptosis, and TLR signaling, were up-regulated in *Mtb*Δ*whiB3* (1.5-fold, *p* < 0.05), whereas genes that negatively regulate autophagy, such as mTOR signaling, were largely down-regulated in the mutant as compared with WT *M. tuberculosis* (1.5-fold, *p* < 0.05) (Fig. 8 and supplemental Table S2). In agreement with the role of WhiB3 in responding to early increase in phagosomal acidity, we observed a significantly greater impact of WhiB3 loss on host transcriptome at an early time point (*i.e.* 12 h postinfection) (Fig. 8). Finally, using qRT-PCR, we validated microarray data by measuring the expression of a selected set of genes involved in phagosomal maturation in a *whiB3*-dependent manner (data not shown). Altogether, the data indicate that WhiB3 plays an important role in influencing expression of host-directed mechanisms associated with controlling intraphagosomal survival of *M. tuberculosis*.

##### WhiB3 Regulates Survival of M. tuberculosis in Vivo

**FIGURE 9.**
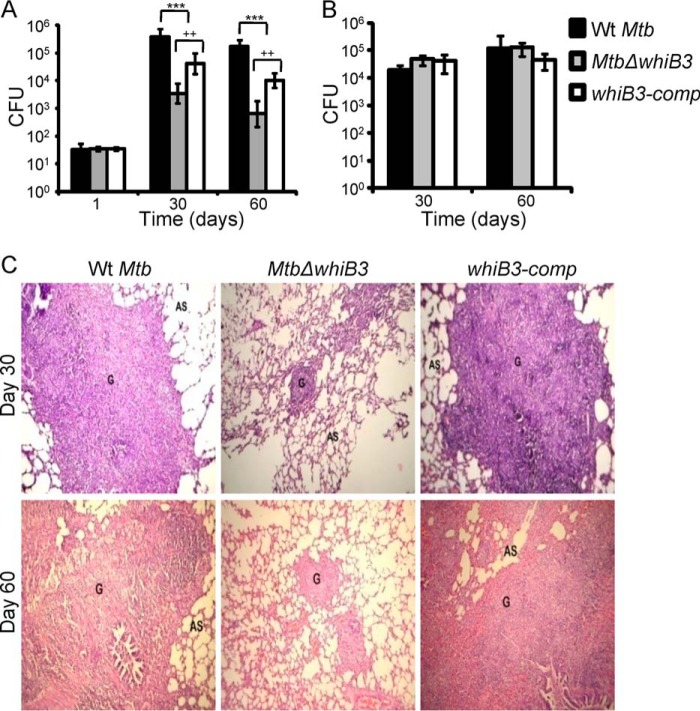
**WhiB3 regulates survival of *M. tuberculosis in vivo*.** Outbred Hartley guinea pigs (*n* = 5) were given an aerosol challenge with WT *M. tuberculosis*, *Mtb*Δ*whiB3*, and *whiB3-comp* strains and assessed for survival in lungs (*A*) and spleen (*B*). *Error bars*, S.D. Statistical significance for the pulmonic and splenic bacterial load was obtained by comparing different strains: ***, *p* < 0.001 (as compared with WT *M. tuberculosis*); ++, *p* < 0.005 (as compared with *whiB3Comp*). *C*, hematoxylin and eosin-stained lung sections (30 and 60 days postinfection) from guinea pigs infected with WT *M. tuberculosis*, *Mtb*Δ*whiB3*, and the *whiB3-comp* strains. The pathology sections show granulomas (*G*) and alveolar space (*AS*). All images were taken at ×40 magnification.

Given that WhiB3 regulates pH-dependent modulation of expression and redox signaling, resulting in differential intracellular trafficking and survival inside macrophages, we hypothesize that WhiB3 plays an important role during *M. tuberculosis* infection. To examine this, we assessed the *in vivo* phenotype of *Mtb*Δ*whiB3* in guinea pigs. Aerosol infection of out-bred Hartley guinea pigs showed a clear growth attenuation of *Mtb*Δ*whiB3* as compared with WT *M. tuberculosis* in the lungs of animals. At day 1 postinfection, cfu analysis showed that nearly identical numbers of bacteria were implanted in the lungs of guinea pigs infected with WT *M. tuberculosis*, *Mtb*Δ*whiB3*, and *whiB3-comp* strain (Fig. 9*A*). At days 30 and 60 postinfection, the number of bacteria present in lungs of animals infected with *Mtb*Δ*whiB3* was ∼70 (*p* = 0.0073) and ∼200-fold (*p* = 0.0007) lower than in those infected with WT *M. tuberculosis*, respectively (Fig. 9*A*). Interestingly, in contrast to our lung data, bacterial numbers in the spleen at days 30 and 60 postinfection were comparable in WT *M. tuberculosis* and *Mtb*Δ*whiB3* (Fig. 9*B*). The attenuated virulence phenotype exhibited by *Mtb*Δ*whiB3* was partially restored in the animals infected with *whiB3-comp* strain (Fig. 9*A*). Histopathological analysis of lungs from infected animals further validated the requirement of WhiB3 during infection. The lungs of *Mtb*Δ*whiB3-*infected animals showed less severe pathology as indicated by decreased tissue consolidation, smaller granulomas, and open alveolar space as compared with the WT *M. tuberculosis* and *whiB3-comp* strains (Fig. 9*C*).

## Discussion

In 1905, Metchnikoff (32) reported the presence of acidic milieu within the phagosomes of macrophages infected with pathogens. Despite this early observation, how *M. tuberculosis* bacilli respond, resist, and persist in response to a gradient of acidic pH during infection remains poorly characterized. Here, we identified acidic pH as a physiological stimulus to which WhiB3 regulates (i) gene expression, (ii) mycothiol redox homeostasis, (iii) phagosomal maturation, and (iv) virulence.

Our microarray data highlight the role of WhiB3 in regulating acid stress response in *M. tuberculosis*. Differential regulation of several genes involved in redox metabolism of *M. tuberculosis* in response to acid stress in a WhiB3-dependent manner and acute sensitivity displayed by *Mtb*Δ*whiB3* at pH 4.5 suggest that WhiB3 facilitates *M. tuberculosis* persistence in response to acid stress by maintaining intramycobacterial redox homeostasis. Until now, direct evidence linking acidic stress encountered in phagosomes to internal redox balance of *M. tuberculosis* was lacking. A recent *in vitro* study, using a genetically encoded redox biosensor (roGFP-R12), has demonstrated that the cytoplasmic redox state of *M. tuberculosis* shifts to reductive when bacilli are cultured under specific carbon sources at pH 5.5 (33). Because conventional roGFPs, such as roGFP-R12, predominantly interact with glutathione redox buffer (34, 35), which is absent in mycobacteria, the utility of roGFPs in *M. tuberculosis* is limited by unknown specificity and poor response to changes in redox potential (15). Moreover, reliable measurement range for roGFPs (*i.e.* between 10 and 90% of sensor oxidation) covers about ±30 mV from the standard midpoint potential (34). Therefore, roGFP-R12 with a less negative midpoint potential (−265 mV) cannot accurately measure a reductive shift in redox potential beyond −295 mV. In this context, Mrx1-roGFP2 with a midpoint potential of −280 mV allowed dynamic and precise imaging of the *E_MSH_* of *M. tuberculosis*, in response to both oxidative and reductive stresses, with high sensitivity and temporal resolution (15, 36). Using this bioprobe, we provide accurate numerical evidence that acidic pH promotes reductive shift in *E_MSH_* of *M. tuberculosis in vitro* and inside phagosomes in a WhiB3-dependent manner. The reductive shift in *E_MSH_* at initial phases of intramacrophage growth is consistent with the rapid drop in vacuolar pH within minutes of infection with *M. tuberculosis* (24), which also serves as an early cue to induce expression of genes linked to reductive stress (*e.g. whiB3*, *whiB7*, and *dosR*) (29, 37). More importantly, inhibition of vacuolar acidification resulted in both the loss of gene expression (28) and a substantial decrease in the *M. tuberculosis* subpopulations with *E_MSH_* reduced.

In contrast to WT *M. tuberculosis*, which preferentially resides in early endosomes, we observed that *Mtb*Δ*whiB3* mainly localized to acidified lysosomes and displayed a higher proportion of *E_MSH_*-oxidized bacilli. These findings support our earlier observations that lysosomes enrich *E_MSH_*-oxidized bacteria, whereas phagosomes with limited acidity (early endosomes) induce a reductive shift in *E_MSH_* of *M. tuberculosis* (15). Interestingly, whereas treatment with BafA1 prevented acidification of *Mtb*Δ*whiB3-*containing phagosomes and reversed intramacrophage survival defect, the proportion of mutant bacilli with *E_MSH_* oxidized remained uninfluenced. One likely possibility is that the loss of WhiB3 compromised the ability of the mutant to respond to changes in the phagosomal environment via the mycothiol redox system. In line with this, several components of the mycothiol pathway, including MSH disulfide reductase involved in recycling MSSM to MSH, are down-regulated in *Mtb*Δ*whiB3* upon acid stress. Alternatively, *Mtb*Δ*whiB3* may exploit another antioxidant system, such as ergothionine (ERG), to respond to the intraphagosomal environment. A compensatory protective role of ERG has already been established in mycothiol-defective mycobacterial strains (38). Because Mrx1-roGFP2 does not respond to ERG (15), further work testing the role of ERG redox potential will allow more precise determination of relative contributions of ERG and MSH pathways in responding to the phagosomal milieu.

The acidic pH-induced reductive *E_MSH_* in WT *M. tuberculosis* most likely resulted from increased synthesis of MSH or a higher rate of MSSM reduction to MSH via the activity of NADPH-dependent MSH disulfide reductase. Expression data indicated a significant up-regulation of MSH-biosynthetic genes in response to acidic pH. Additionally, a decreased expression of respiratory genes involved in regenerating NAD^+^ cofactor (*e.g. nuo* operon), along with the up-regulation of fatty acid catabolic genes, which generate excessive NADH through β-oxidation, may further lead to accumulation of NADH/NADPH cofactors during acidity. Because excessive accumulation of NADH/NADPH is known to elevate endogenous ROS levels through autoxidation or via Fenton reaction (39, 40), a reductive shift in *E_MSH_* by NADPH-dependent conversion of MSSM to MSH via MSH disulfide reductase could be a mechanism to dispose of excess reductants. In this context, studies in *M. tuberculosis* have indicated only a marginal increase in the NADPH pool at acidic pH, whereas levels of cytoplasmic thiols, such as MSH and CoA-SH, were substantially elevated (33, 41), agreeing with our findings that the MSH pathway can function as a reductive sink to reduce toxicity associated with low pH. Further strengthening this connection is our finding showing the inability of *Mtb*Δ*whiB3* to maintain *E_MSH_* reduced, along with a previous report demonstrating massive accumulation of NADH/NADPH in *Mtb*Δ*whiB3* inside macrophages (17). The mycothiol redox system has recently been shown to interact with a major antioxidant enzyme; superoxide dismutase, to exert an efficient adaptive response during infection (42). In light of this, an early elevation in reductive capacity of mycothiol (*E_MSH_* reduced), in response to vacuolar pH, might be important to activate additional mechanisms to detoxify a range of hostile radicals that *M. tuberculosis* encounters later in the infection cycle (*e.g.* during immune activation). Altogether, these observations, along with the fact that external acidity does not increase H^+^ concentration inside *M. tuberculosis* (10), serve to implicate intrabacterial reductive *E_MSH_* as an internal physiological signal to which *M. tuberculosis* responds through WhiB3 to coordinate gene expression and survival. These findings were further strengthened by our data showing a complete loss of *whiB3* induction along with other pH-responsive genes in an *M. tuberculosis* strain lacking mycothiol (*Mtb*Δ*mshA*) at acidic pH. Because WhiB3 is cytoplasmically located and implicated in responding to changes in intracellular redox conditions through redox-sensitive [4Fe-4S] cluster (13, 17), we hypothesize that the intramycobacterial reductive *E_MSH_* under acidic stress can promote accumulation and/or stabilization of the reduced form of the 4Fe-4S cluster ([4Fe-4S]^1+^-WhiB3), which directly or indirectly activates pH-responsive genes in *M. tuberculosis*. Alternatively, because the DNA binding activity of apo-WhiB3 (without an Fe-S cluster) is modulated by the redox state of its cysteine thiols (17), an interesting possibility is that the reversible *S*-mycothiolation of WhiB3 thiols in response to pH-mediated changes in *E_MSH_* may function as a redox-regulatory switch to modulate gene expression. The functional linkage between MSH and WhiB3, as revealed in this study, can now be exploited to understand the molecular mechanism(s) of how mycothiol exerts its influence on the redox behavior and gene regulatory properties of WhiB3.

**FIGURE 10.**
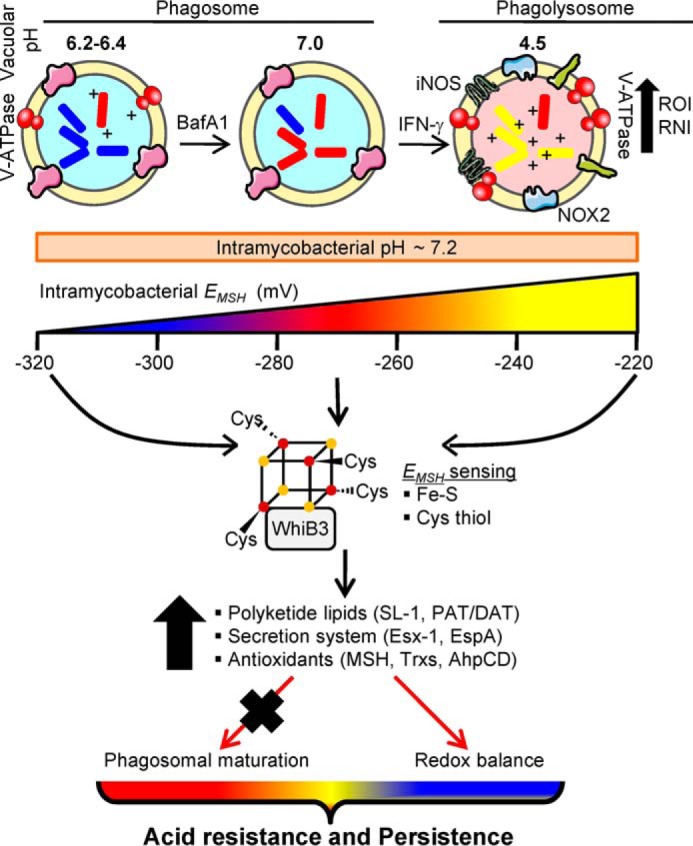
**Model depicting the role of *E_MSH_* and WhiB3 in responding to acid stress during infection.** In resting macrophages, *M. tuberculosis* impairs phagosome maturation and preferentially resides in a mildly acidic environment. Activation with IFN-γ induces phagosomal-lysosomal fusion to elevate the levels of proton (pH 4.5), reactive oxygen intermediates (*ROI*), and reactive nitrogen intermediates (*RNI*) in the microenvironment. Despite these changes in external pH, the internal pH of *M. tuberculosis* remains close to neutral (pH 7.2). Importantly, these variations in phagosomal pH induce dynamic changes in the *E_MSH_* of *M. tuberculosis*. Limited acidification inside resting macrophages induces a reductive shift in *E_MSH_* of *M. tuberculosis* (approximately −305 mV), whereas activation of macrophages induces oxidative shift (−240 mV). Pharmacological inhibition of phagosomal acidification by BafA1 neutralizes acidic pH to prevent reductive shift in *E_MSH_* of *M. tuberculosis. M. tuberculosis* responds to phagosomal acidification with the help of a putative redox-sensitive transcription factor, WhiB3. WhiB3 can sense changes in intrabacterial *E_MSH_* via its Fe-S cluster or cysteine thiols (*S*-mycothiolation) to modulate the expression of virulence genes involved in blocking phagosomal maturation (*e.g.* polyketides, secretory antigens) and redox homeostasis. Impaired ability of *Mtb*Δ*whiB3* to maintain mycothiol balance and block phagosomal maturation, along with the survival defect *in vivo*, suggests a central role of WhiB3 in regulating mycobacterial persistence in response to acid stress. The exact mechanisms by which WhiB3 senses pH-induced changes in internal *E_MSH_* via 4Fe-4S and/or cysteine thiols remain to be identified.

We found that *Mtb*Δ*whiB3* has an impaired ability to arrest phagosomal maturation, which can be rescued by supplementation of surface-associated polyketide lipids from WT *M. tuberculosis*. These discoveries have significant implications in understanding mycobacterial pathogenesis, which suggests an intertwined association between immunomodulatory virulence factors and the core metabolic processes in *M. tuberculosis*. We propose that WhiB3-mediated synthesis of virulence factors, including secretory lipids and proteins (ESX-1 system), in response to intracellular redox changes associated with acidic pH will play a larger part, in both redox maintenance and in counteracting phagosomal acidification, to ensure long term persistence of *M. tuberculosis* (Fig. 10). Although our results indicate that WhiB3 is a major regulator of pH and redox homeostasis, other regulators, such as PhoP, that similarly modulate the expression of virulence factors to influence phagosomal maturation and intraphagosomal survival can provide overlapping control over pH and redox response in *M. tuberculosis* (33, 43). Finally, the physiological significance of our mechanistic findings comes from dramatic attenuation of *Mtb*Δ*whiB3* in the lungs of guinea pigs. Our results are in complete contrast with the previous findings on the *Mtb*Δ*whiB3* phenotype *in vivo* (11, 44). It is possible that hypoxic caseous TB granulomas formed in lungs of guinea pigs expose *M. tuberculosis* to a more acidic environment (45), which might have necessitated the need for a WhiB3-dependent redox sensing pathway for ensuring redox homeostasis, survival, and persistence of *M. tuberculosis in vivo*.

In summary, we have identified a new mechanism exploited by *M. tuberculosis* to respond to phagosomal pH. Our study provides a unique example of the tight connections forged between core redox machinery and virulence in mycobacterial pathogenesis. Furthermore, pH-induced redox signalling and its connection with gene expression and virulence may be relevant to other intracellular pathogens. For example, acidification is required for virulence expression in *Salmonella* (46), phagosomal escape of *Listeria monocytogenes* (47), and efficient replication of *Legionella pneumophilla* and *Coxiella burnetti* (48, 49). Thus, our findings have broad implications for several intracellular pathogens for which phagosomal pH plays a critical role in modulating virulence and long term persistence.

## Author Contributions

M. M. and A. S. designed the research; M. M. and R. S. R. performed research; and M. M. and A. S. wrote the paper. All authors analyzed the results and approved the final version of the manuscript.

## Supplementary Material

Supplemental Data

## Footnotes

3 The abbreviations used are:

*E_MSH_*: mycothiol redox potential
MSH: mycothiol
BafA1: bafilomycin A1
CCCP: carbonyl cyanide *m*-chlorophenylhydrazone
ERG: ergothionine
V-ATPase: vacuolar H^+^-ATPase
Mrx1: mycoredoxin
MOI: multiplicity of infection
PMA: phorbol 12-myristate 13-acetate
qRT-PCR: quantitative RT-PCR
roGFP: redox-sensitive green fluorescent protein
ROS: reactive oxygen species
RNS: reactive nitrogen species.
