# Supplementary material for: Mycobacterium tuberculosis WhiB3 Responds to Vacuolar pH-induced Changes in Mycothiol Redox Potential to Modulate Phagosomal Maturation and Virulence
Source: J Biol Chem. 2015 Dec 4;291(6):2888–903. doi: 10.1074/jbc.M115.684597 (PMC4742752; doi:10.1074/jbc.M115.684597)
Supplement: Supplemental Data [file supp_291_6_2888__index.html]

Mycobacterium tuberculosis WhiB3 responds to vacuolar pH- induced changes in mycothiol redox potential to modulate phagosomal maturation and virulence — Mycobacterium tuberculosis WhiB3 Responds to Vacuolar pH-induced Changes in Mycothiol Redox Potential to Modulate Phagosomal Maturation and Virulence — Redox-mediated Acid Resistance in M. tuberculosis — Supplemental Data 

# *Mycobacterium tuberculosis* WhiB3 Responds to Vacuolar pH-induced Changes in Mycothiol Redox Potential to Modulate Phagosomal Maturation and Virulence

## Supplemental Data

- Table S1 (.xls, 379 KB) - Raw microarray excel data sheet (wt Mtb and WhiB3 mutant)
- Table S2 (.xls, 336 KB) - Raw microarray excel data sheet (THP-1)
